# Understanding the Surprising Oxidation Chemistry of Au−OH Complexes

**DOI:** 10.1002/cphc.202200475

**Published:** 2022-10-13

**Authors:** Silène Engbers, Johannes E. M. N. Klein

**Affiliations:** ^1^ Molecular Inorganic Chemistry Stratingh Institute for Chemistry Faculty of Science and Engineering University of Groningen Nijenborgh 4 9747 AG Groningen The Netherlands

**Keywords:** gold hydroxides, mechanisms, oxidation, oxygen atom transfer, proton coupled electron transfer

## Abstract

Au is known to be fairly redox inactive (in catalysis) and bind oxygen adducts only quite weakly. It is thus rather surprising that stable Au−OH complexes can be synthesized and used as oxidants for both one‐ and two‐electron oxidations. A charged Au^III^−OH complex has been shown to cleave C−H and O−H bonds homolytically, resulting in a one‐electron reduction of the metal center. Contrasting this, a neutral Au^III^−OH complex performs oxygen atom transfer to phosphines, resulting in a two‐electron reduction of the hydroxide proton to form a Au^III^−H rather than causing a change in oxidation state of the metal. We explore the details of these two examples and draw comparisons to the more conventional reactivity exhibited by Au^I^−OH. Although the current scope of known Au−OH oxidation chemistry is still in its infancy, the current literature exemplifies the unique properties of Au chemistry and shows promise for future findings in the field.

## Graphical abstract

**Au is known** to be rather redox‐inactive in catalysis with molecular catalysts and only binds oxygen weakly. Nevertheless, examples of Au−OH complexes in both the formal +I and +III oxidation states have recently been reported. Surprisingly, formal Au^III^−OH complexes have shown to be promising oxidants for oxygen atom transfer and proton‐coupled electron transfer reactions. In this concept article the authors describe the known subtilities of these reactions and outline future possibilities for the field.

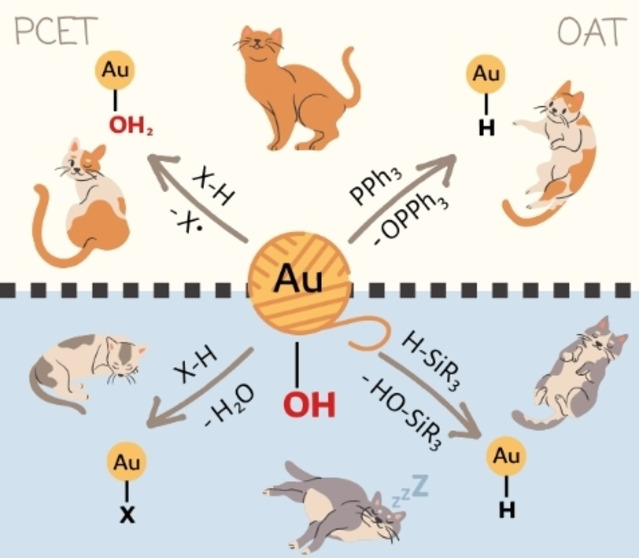

## Introduction

Gold is known to have a particularly weak M−OH bond, the weakest within the coinage metals,^[1]^ and is the metal with the smallest hydroxide binding energy to its surface.^[2]^ We can thus consider Au to be among the least oxophilic metals. In addition, Au is known to have rather high redox potentials, making it quite resistant to oxidation state changes.^[3]^ Nevertheless, gold nanoparticles have been identified as promising oxidants for the industrial scale oxidation of propylene.^[4]^ Though the mechanism for such transformations is as of yet not fully understood, the involvement of an active Au−OH species has been deemed unlikely.^[5]^ Contrasting this, Au−OH bonds have been reported to form on gold electrode surfaces and play a crucial role in the electrochemical oxygen evolution reaction.^[6]^ Hence, electrochemical activation appears to be a key element for heterogeneous Au−OH to act as an oxidant.

**Figure 1:**
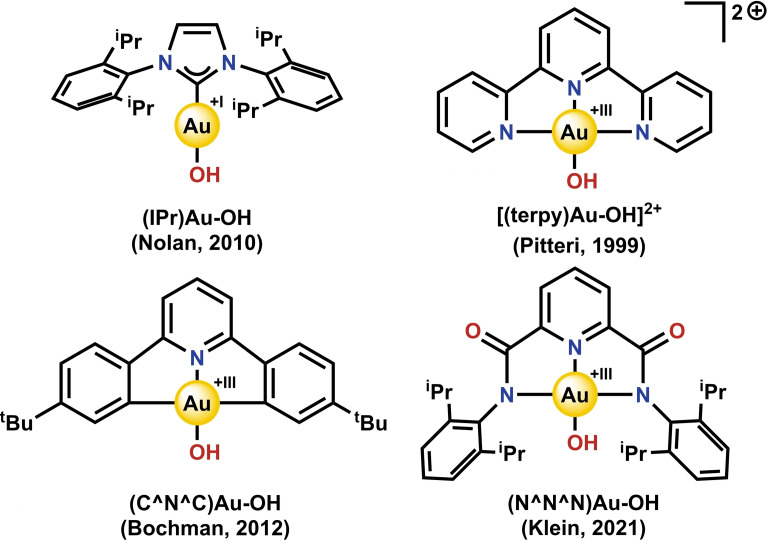
Examples of Au−OH complexes for which oxidation reactivity studies have been performed.[^9a^, ^9c^, ^9d^, ^9m^]

The intrinsically weak nature of the Au−OH bond suggests that molecular Au−OH complexes may be unstable and synthetically challenging to obtain. Though the simple Au^I^(OH) salt requires high temperatures and pressures to form,^[7]^ Au^III^(OH)_3_ is well known and commercially available.^[8]^ This contrast may be attributed to an increased hardness of Au in the formal +III oxidation state. Some examples of structurally well‐defined Au−OH complexes in both the formal +I and +III oxidation states have emerged in the literature over the last few decades (for selected crucial examples see Figure 1).^[9]^ Commonly Au^I^−OH complexes are linear and, to the best of our knowledge, exclusively bear *N*‐heterocyclic carbene (NHC) ligands.[^9c^, ^9e^, ^9f^, ^9g^, ^9h^, ^9i^] Au^III^−OH complexes are square planar with the vast majority bearing pincer‐type ligands.[^9a^, ^9b^, ^9d^, ^9i^, ^9j^, ^9k^, ^9m^] Both these classes of compounds have been shown to exhibit diverse reactivity, encompassing Brønsted base‐type reactivity,[^9d^, ^9i^, ^9j^, ^10^] transmetallations,[^9c^, ^9d^, ^9j^, ^11^] and insertions of CO or CO_2_ into the Au−OH bond.^[12]^ Curiously, some Au^III^−OH complexes have recently been shown to also perform oxidations *via* proton coupled electron transfer (PCET)^[13]^ and oxygen atom transfer (OAT) reactions.[^9m^, ^14^]

## Proton Coupled Electron Transfer (PCET)

**Scheme 1:**
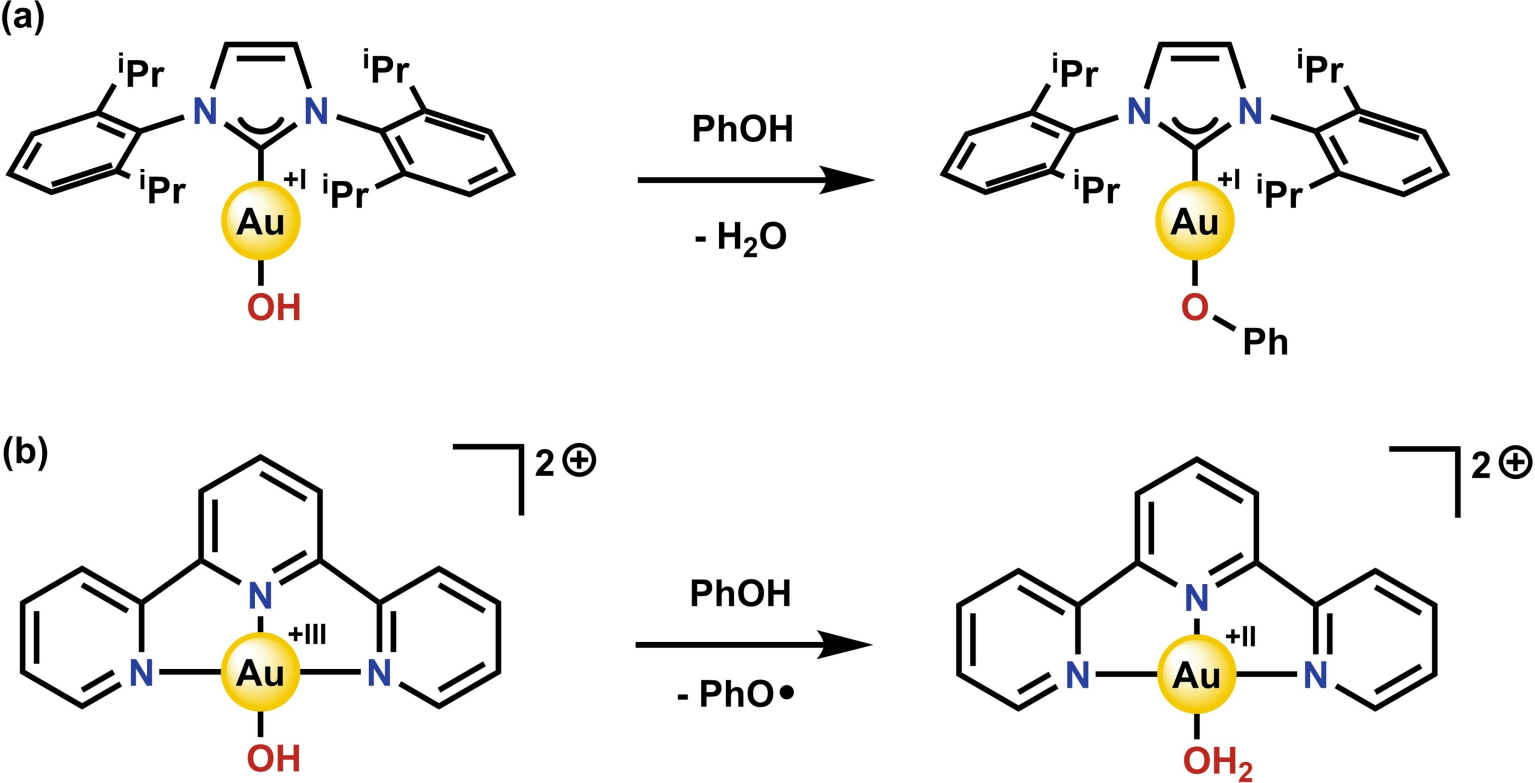
Reported reactivity of formal (**a**) Au(I) and (**b**) Au(III) complexes with phenol.[^10c^, ^13a^]

The Brønsted basicity of Au−OH complexes has made them convenient synthons for the preparation of more synthetically challenging Au complexes.[^9d^, ^9j^, ^10^] Protonation of the hydroxide ligand forms a labile aqua ligand which will readily exchange for the conjugate base of the acid employed. If the acid is carefully chosen, the desired complex can thus be synthesized cleanly, with only water being produced as waste/side product. Although a variety of formal Au(I) and Au(III) complexes have been employed for this, (IPr)Au−OH, which can deprotonate acids with a *p*K_a_<30,^[10a]^ has received the most attention. It was further shown that acids for which the conjugate acid is non‐coordinating will conveniently yield (IPr)Au^+^, a common active species in Au catalysis.^[15]^ (IPr)Au−OH has thus found many applications as a pre‐catalyst in silver‐free protocols.^[16]^ To the best of our knowledge though, no Au^I^−OH complex has been reported to combine deprotonation with electron transfer. Moreover, (IPr)Au−OH will cleanly deprotonate phenols, which are commonly used substrates for PCET, to form (IPr)Au−OAr where no electron transfer from the substrate has occurred and the oxidation state of the metal is unaltered (Scheme 1a).^[10c]^

In contrast, [(terpy)Au−OH]^2+^ was recently found to cleave the weak C−H bonds of 1,4‐cyclohexadiene (CHD) and 9,10‐dihydroanthracene (DHA), as well as the O−H bonds of a variety of phenols homolytically (Scheme 1b).[^13a^, ^13b^] Although various metal hydroxides are known to perform PCET reactions,^[17]^ such reactivity is surprising for formal Au(III) due to the formation of mononuclear Au(II) species, which are rare and known to be highly unstable.^[18]^ It should be noted that the formation of PCET products will not necessarily provide the driving force for the reaction.^[13c]^ Further reactivity occurs after the initial PCET step: [(terpy)Au^II^−OH_2_]^2+^ decomposes immediately upon formation, CHD and DHA undergo a second PCET step to provide benzene and anthracene, respectively, and the phenoxy radicals will react further by either dimerizing or reacting with water to form quinones.^[13a]^

**Scheme 2:**
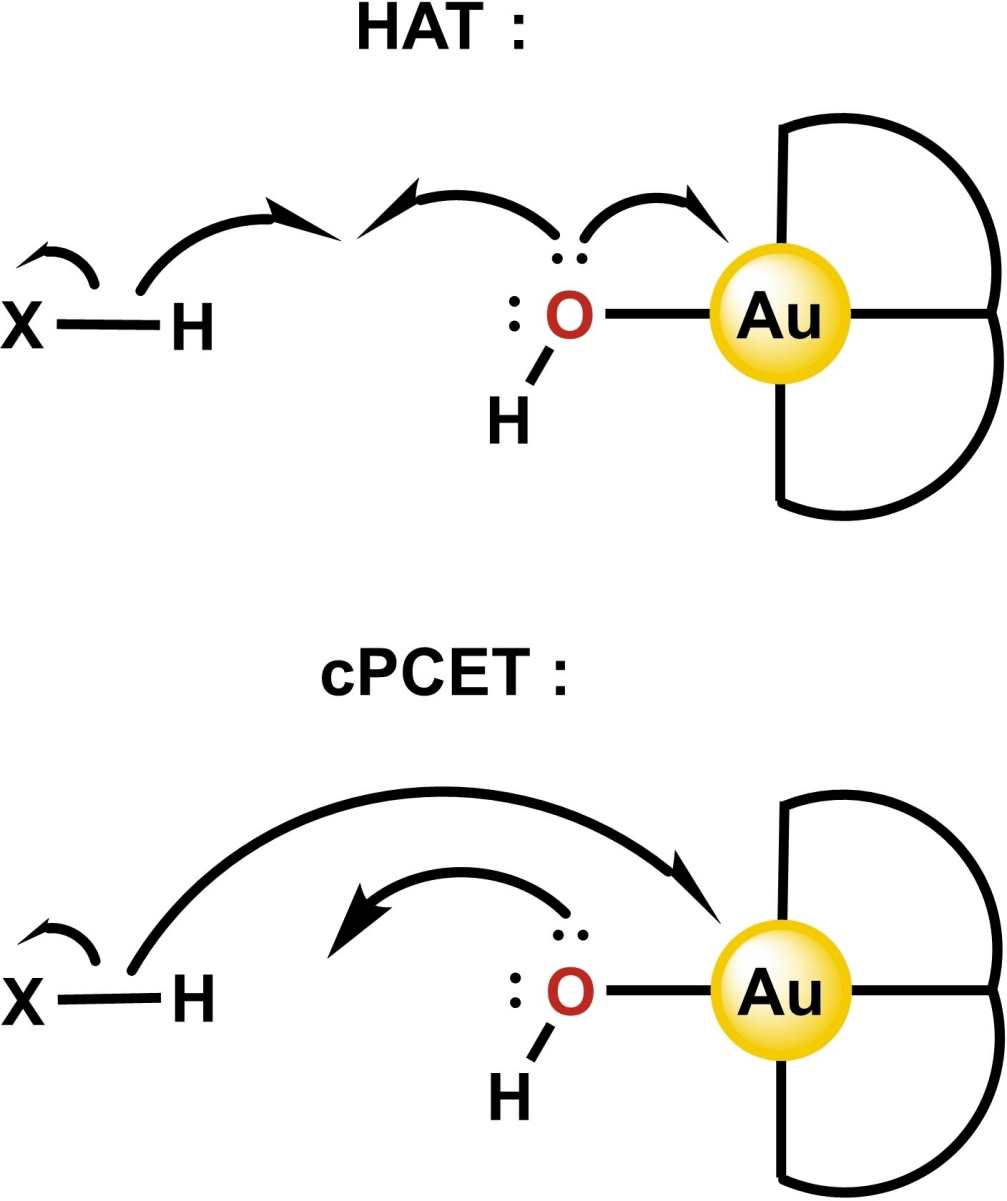
Curly arrow representations of the electron flow in the HAT and cPCET mechanisms. Adapted from Ref. [13c].

Kinetic data indicated that PCET for both C−H and O−H bond breaking by [(terpy)Au−OH]^2+^ occurs *via* a concerted reaction mechanism.^[13a]^ Within the concerted regime, two mechanistic scenarios are possible.^[19]^ Let us briefly define the two terms used herein. The first is hydrogen atom transfer (HAT), in which the proton and electron are transferred together as one entity (hydrogen atom) to the hydroxide moiety of the Au−OH complex (Scheme 2). The second is concerted proton coupled electron transfer (cPCET), where the electron and proton move simultaneously but as two separate entities, the proton travelling to the hydroxide moiety of the Au−OH complex and the electron directly to the Au atom (Scheme 2).

A computational study on the C−H and O−H bond cleavage reactions of CHD and phenol by [(terpy)Au−OH]^2+^ elucidated that cPCET is operative in both cases.^[13c]^ As a point of comparison, (N^N^N)Cu^III^−OH is known to cleave C−H bonds *via* HAT and O−H bonds *via* cPCET.^[19d]^ The fact that the Au atom in [(terpy)Au−OH]^2+^ accepts the electron directly from the substrate in both cases is thus rather surprising, and suggests that Au(III) is actually quite easily reduced. Indeed, [(terpy)Au−OH]^2+^ undergoes an irreversible reduction at a potential of only −0.13 V vs. ferrocene/ferrocenium (Fc/Fc^+^).^[13a]^ This is much lower than that of the neutral (C^C^C)Au−OH complex, which undergoes an irreversible reduction at −2.39 V vs. Fc/Fc^+^.^[20]^ (C^C^C)Au−OH is still capable of being reduced though as it forms Au^II^‐Au^II^ dimers upon reaction with (C^C^C)Au−H.[^11a^, ^20^] Hence, in contrast to traditional belief, charged Au^III^−OH complexes can be viable one‐electron oxidants.

## Oxygen Atom Transfer (OAT)

**Scheme 3:**
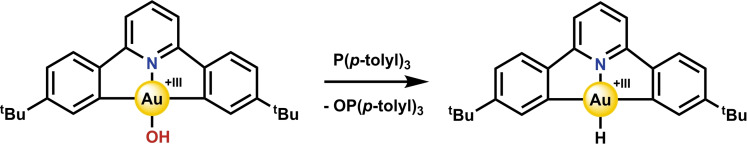
OAT reactivity of (C^C^C)Au−OH.^[14a]^

Whereas PCET is a one‐electron process, OAT entails a two electron oxidation.^[21]^ OAT from a metal complex is thus usually associated with a two‐fold reduction of the metal center.^[22]^ Due to the particularly large redox potential of the Au(I)/Au(III) pair,^[23]^ OAT could be considered unlikely for formal Au(III). Remarkably though, (C^C^C)Au−OH was actually shown to perform OAT to a variety of phosphines without any change in the formal oxidation state of Au (Scheme 3).^[14a]^ (C^C^C)Au−H is generated, a stable compound often synthesized by transmetallation with hydrosilanes.^[11a]^ During OAT, the hydride is produced by reduction of the hydroxide proton, revealing that H^+^ is more easily reduced than formal Au(III) in this case.

It is interesting to note that (C^C^C)Au−OOH, (C^C^C)Au−OO^t^Bu, (C^C^C)Au−OMe, (IPr)Au−OO^t^Bu and (IPr)Au−OOH all similarly perform OAT without change in the formal oxidation state of Au.[^14^, ^24^] However, attempts at reproducing this with (IPr)Au−OH were unsuccessful, as no reaction was observed with phosphines.^[14b]^ The root cause of this lack of reactivity cannot be ascribed to instability of (IPr)Au−H, as this is a known compound which can be synthesized from (IPr)Au−OH by transmetallation with hydrosilanes.^[9c]^ We may thus consider the OAT reactivity of Au−OH complexes to be constrained to formal Au(III) complexes.

Much curiosity has arisen on the mechanism by which Au^III^−OH complexes perform OAT. Reacting (C^C^C)Au−OD with tri(*p*‐tolyl)phosphine revealed a kinetic isotope effect of 1.45, consistent with an O−H bending mode.^[14a]^ Based on this, a concerted mechanism was proposed wherein the phosphine directly attacks the oxygen, and the H atom simultaneously navigates to Au.^[14a]^ The reaction of (C^C^C)Au−OMe with phosphines similarly reduces the methyl group of the methoxide to form the formal Au(III) complex (C^C^C)Au−Me.^[24a]^ However, this reaction proceeds over several days at room temperature instead of the few hours at −40 °C required for the hydroxide complex.[^14a^, ^24a^] Computationally, a transition state involving direct attack of the oxygen atom was found for the reaction of (C^C^C)Au−OMe with triphenylphosphine,^[24]^ similar to that proposed for (C^C^C)Au−OH.^[14a]^ Its barrier is computed to be high though (ΔG^≠^=39.7 kcal mol^−1^),^[24a]^ indicating that alternative pathways should probably be considered.

The equally neutral (N^N^N)Au−OH complex was found to have very similar intrinsic bond orbital partial charge distributions across the Au−O bond compared to that of (C^C^C)Au−OH (0.484/1.469 and 0.510/1.447, respectively, indicating a rather electron deficient oxygen atom).^[9m]^ Hence, these two complexes might be expected to exhibit similar reactivity. Surprisingly, (N^N^N)Au−OH is unreactive towards OAT with phosphines at room temperature.^[9m]^ The most prominent difference between the two complexes is their steric footprint around the Au atom. Whereas the (C^C^C) ligand is completely flat in close proximity to Au, the (N^N^N) ligand has its aryl groups oriented perpendicularly to the central pyridine ring. Buried volume calculations show that Au is indeed substantially more sheltered in (N^N^N)Au−OH (79.3 compared to 62.9 %).^[9m]^ It was hence suggested that the mechanism requires an Au−P interaction, which is possible for (C^C^C)Au−OH, but sterically inhibited for (N^N^N)Au−OH.^[9m]^ However, further investigation is necessary in order to fully uncover the subtleties of this reaction mechanism.

## Conclusion

Despite gold's adversity to hydroxide ligands and oxidation state changes, some Au−OH complexes are capable of performing either one‐electron or two‐electron oxidations (PCET and OAT, respectively). Relatively unsurprisingly, this reactivity seems limited to complexes in the higher oxidation state of formal +3, as the popular (IPr)Au−OH complex deprotonates phenol without accepting an electron and is unreactive to oxygen transfer to tri(*p*‐tolyl)phosphine. So far, PCET has only been shown to occur with the charged [(terpy)Au−OH]^2+^ and OAT only with the neutral (C^C^C)Au−OH. Considering the much more accessible redox potential of [(terpy)Au−OH]^2+^ compared to (C^C^C)Au−OH, it is actually rather logical that the former is singly reduced at the metal center whereas the latter diverts the double reduction to the hydroxide proton. However, in order to fully understand the origin of reactivity and subtle differences between complexes, we encourage future work to report and compare the redox potentials, pK_a_s, and HOMO/LUMO gaps.

Although catalytic transformations involving oxidation state changes based on gold were deemed inaccessible for a long time, more recently elementary organometallic steps have become possible.^[25]^ These examples are, however, limited to steps that involve oxidation state changes due to transfer of electron pairs. Gold‐based photoredox catalysis has emerged as a research field wherein changes between the oxidation states +I and +III have been realized with previously unprecedented ease.[^25b^, ^26^] This includes the proposal of the involvement of transient Au(II) species, and hence the involvement of single‐electron transfer reactions.[^25b^, ^26^] Considering that Au^III^−OH species can perform both one and two electron oxidations of substrates thermally, as outlined above, we envision further possibilities for the development of gold‐catalyzed transformations that feature transient Au−OH intermediates.^[27]^ Much remains to be explored regarding the scope of Au−OH complexes as oxidants as well as the mechanisms through which these transformations occur. We hope that the brief outlook provided here will spark interest in the field and lead to new discoveries in this area.

## Conflict of interest

The authors declare no conflict of interest.

1

## Data Availability

Data sharing is not applicable to this article as no new data were created or analyzed in this study.
